# Systematic review of surgical regenerative treatment for apicomarginal lesions in periapical surgery

**DOI:** 10.4317/medoral.26405

**Published:** 2024-04-14

**Authors:** Lina Attar-Attar, Juan Carlos Bernabeu-Mira, Juan Cervera-Ballester, Miguel Peñarrocha-Diago, María Peñarrocha-Diago

**Affiliations:** 1 Dentist, University of Valencia, Spain; 2 Predoctoral researcher. Oral Surgery Unit, Faculty of Medicine and Dentistry, University of Valencia, Spain; 3 Master in Oral Surgery and Implantology, Oral Surgery Unit, Faculty of Medicine and Dentistry, University of Valencia, Spain; 4 Professor of Oral Surgery. Director of the Master's Program in Oral Surgery and Implantology, Faculty of Medicine and Dentistry, University of Valencia, Spain; 5 Professor of Oral Surgery. Faculty of Medicine and Dentistry, University of Valencia, Spain

## Abstract

**Background:**

Apicomarginal lesions affect the root apex and root surface concurrently and reduce the success rate in periapical surgery. The purpose of this systematic review was to analyze the published literature on the surgical treatment of apicomarginal lesions in periapical surgery.

**Material and Methods:**

A systematic review was conducted on PRISMA statement. Three data bases (PubMed-Medline, Scopus, and Embase) were searched up to March 2023. The inclusion criteria for this systematic review encompass studies pertaining to apicomarginal lesions and their surgical treatment, both preclinical and clinical in nature (including randomized trials, prospective, and retrospective observational trials), without any language or time limitations. Exclusion criteria encompass studies with duplicated population data, no description of the surgical treatment or regenerative material. Different tools for the assessment of bias were applied for each study design

**Results:**

A total of 155 articles were searched and 10 were included. Studies on teeth with apicomarginal lesions undergoing periapical surgery showed a high success rate when regenerative techniques were used, resulting in reduced probing depth, increased bone formation on the root surface, increased root cementum formation, and reduced healing by junctional epithelium. Guided tissue regeneration, platelet-rich plasma or fibrin, and enamel matrix derivatives have emerged as alternative treatments offering favorable outcomes.

**Conclusions:**

The use of regenerative materials in periapical surgery could improve the prognosis of apicomarginal lesions. Future research in this field should aim to standardize classification and healing criteria to enhance comparability across studies and provide more conclusive evidence for optimal treatment approaches.

** Key words:**Apicomarginal lesion, periapical surgery, endodontic surgery, bone regeneration, bone graft, barrier membranes.

## Introduction

Periapical surgery is a procedure that preserves teeth by removing lesions around their root apex (1). Success depends on factors like surgical technique, apical resection level, technological equipment, surgeon's experience, and retrograde cavity material (2). Age, gender, and symptoms have minimal impact, but lesion size, furcation involvement, and location affect success rates, with incisors and canines showing better outcomes (3,4,5).

**Figure 1:**
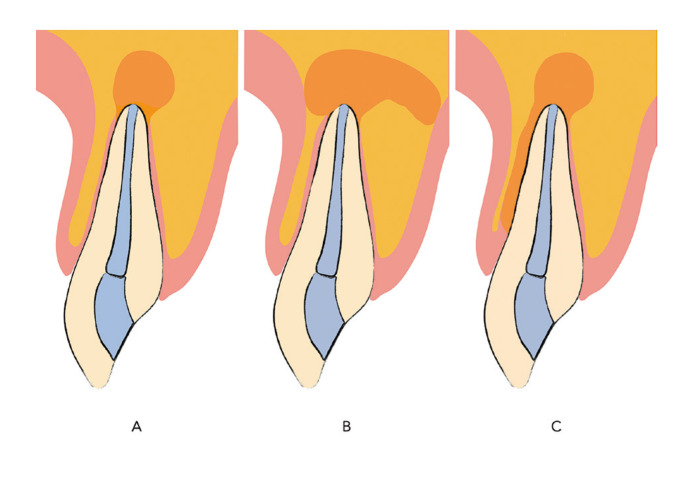
Classification of bone defects according to Von Arx and Cochran. (A) Four-Walled bone defect. (B) Tunnel bone defect. (C) Apicomarginal lesions.

Lesions in periapical surgery are classified into three main types according to Von Arx and Cochran (6): four-walled osseous defects, tunnel-shaped osseous defects, and apicomarginal lesions (Fig. 1). Success rates generally exceed 90% (7), but apicomarginal lesions pose challenges, reducing success to 37% (7) without regenerative techniques and 60% with them (8) due to extended junctional epithelium formation (9). Another classification system by Kim *et al*. (10) distinguished apical lesions of endodontic origin (A, B, C) from those of combined endodontic and periodontal origin (D, E, F). Detritch *et al*. further categorize apicomarginal lesions into Types I, II, III, and IV based on their nature. Regardless of the classification used, all agree that apicomarginal lesions significantly impact the success of periapical surgery. Additionally, Detritch *et al*. (11) considered that the term 'apicomarginal' encompasses a large group of lesions, classifying them into types I, II, III, and IV, with Type I being endoperiodontal lesions, Type II endodontic lesions, Type III osseous dehiscences, and Type IV miscellaneous or mixed lesions.

The presence of apicomarginal lesions is one of the most challenging situations in periapical surgery, particularly when the vestibular bony cortex is completely absent (6). Therefore, their presence might indicate the use of regeneration techniques. Over time, new treatment modalities have emerged that have not yet proven to be superior to the conventional technique of flap replacement over the defect, although apicomarginal lesions could benefit from them, as placing a physical barrier between the root and the epithelium could prevent its growth over the bony defect and allow periodontal ligament and periosteum cells to populate the blood clot and regenerate the lost tissue (5). Further research is needed to explore the potential improvement in healing through the use of regenerative materials since there is limited information and data available regarding regenerative therapy for these types of lesions (9), and there appears to be a lack of controlled clinical and experimental studies evaluating the topic. Techniques such as guided tissue regeneration (GTR), platelet-rich plasma (PRP) or fibrin (PRF), and enamel matrix derivatives (EMD) could enhance the prognosis of periapical surgery in apicomarginal lesions (4,10), but published clinical data on the therapeutic approach are limited.

The purpose of this systematic review is focused on their medium and long-term prognosis of the apicomarginal surgery depending on the type of the material and technique of the regeneration for the periapical surgery. The secondary objective is to update the information available in the literature.

## Material and Methods

An extensive literature search was conducted following the PRISMA (Preferred Reporting Items for Systematic Reviews and Meta-Analysis) guidelines for systematic reviews and meta-analyses, available at https://www.prisma-statement.org// (12).

- PICO Question

The PICO question was: Are regenerative techniques in periapical surgery advantageous for the treatment of patients with apicomarginal lesions compared to the conventional technique of flap replacement over the lesion without the use of regenerative materials?

- Search Strategy

The following databases were consulted: Pub-Med MEDLINE, Scopus, and Embase, between September 2022 and March 2023, using the keywords "apicomarginal," "apicomarginal lesion," "surgery," "periapical surgery," "endodontic surgery," "cortex eroded," "dehiscence," "bone regeneration," "bone graft," and "barrier membranes," combined using Boolean operators such as "AND" and "OR."

- Selection Criteria

The following inclusion and exclusion criteria were applied to select the studies:

Inclusion criteria:

1. Studies focusing on apicomarginal lesions and their surgical treatment.

2. Preclinical studies

3. Clinical studies (randomized trials, prospective or retrospective observational trials).

4. No language or time limitations were applied.

Exclusion criteria:

1. Studies with duplicated population data.

2. No description of the surgical treatment.

3. No description of the regenerative material.

- Study Selection

The identification, screening, and eligibility phases were processed in duplicate by two independent reviewers (LAA and JCB). In case of disagreement between reviewers at any stage, a third reviewer (MPD) was consulted. The Kappa coefficient (k) was used to assess the level of agreement between LAA and JCB at each stage (13). After applying the search strategy, duplicated articles from different databases were removed. Titles and abstracts were reviewed to exclude studies unrelated to the topic of our systematic review. Subsequently, full-text articles were analyzed against the selection criteria.

- Data Extraction

Two independent reviewers (LAA and JCB) separately retrieved the following recorded study variables: author and year of publication, study type, scientific evidence level (according to the SIGN evidence level for intervention studies), type of regeneration, number of cases, pharmacological treatment, previous defect classification (if authors used any previously defined classification in the literature), criteria for healing employed (if authors used any previously defined criteria in the literature), and outcomes obtained.

- Assessment of Bias

The risk of bias analysis was conducted by two independent reviewers (JCBM and JCB) using various analytical tools, depending on the type of included study. The SYRCLE guideline was applied for preclinical animal studies (14), the Cochrane risk of bias tool was used for randomized clinical trials (15), and the ROBINS-I was consulted for non-randomized intervention studies (16). Disagreements between reviewers were resolved through discussion with a third advisor (MPD).

## Results

**Figure 2:**
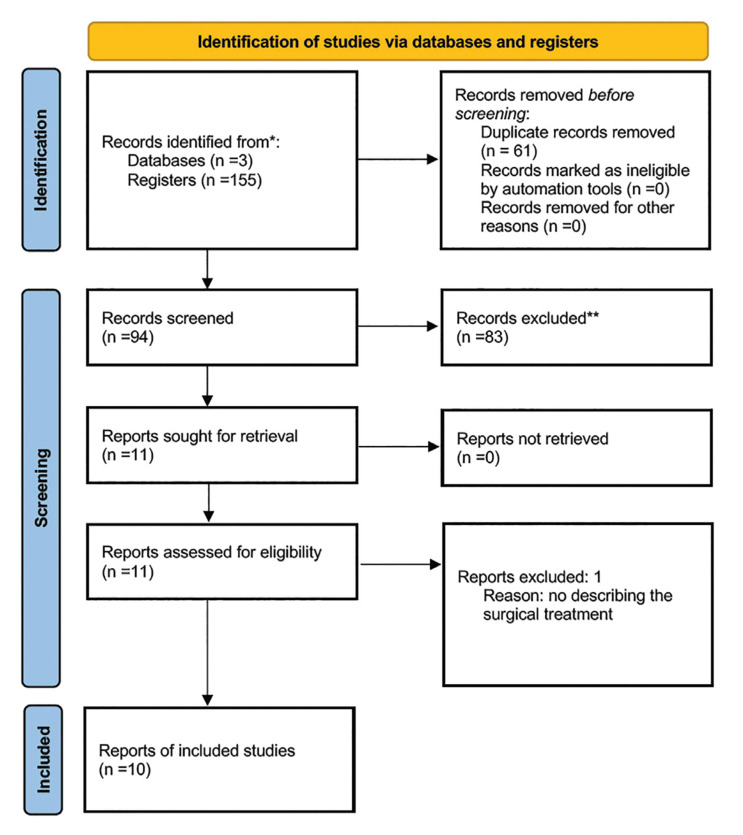
PRISMA 2020 flow diagram for new systematic reviews which included searches of databases and registers only.

A total of 155 articles were identified through the literature search. After removing duplicates, 94 articles underwent title and abstract screening, and 11 were deemed potentially eligible for inclusion in the study. After full-text reading, one study was excluded, resulting in 10 articles (8-10,17-23) being included in the systematic review, based on the selection criteria (Fig. 2).

Cohen's kappa coefficient showed excellent agreement during the identification (k = 1), screening (k = 1), and eligibility phases (k = 1).

Four randomized clinical trials in humans (8,9,17,18), two case series (10,19), one retrospective study (21), and three experimental animal studies (20,22,23) were analyzed. The total sample size of apicomarginal defects treated through surgical intervention was *n* = 271 (Table 1).

**Figure 3:**
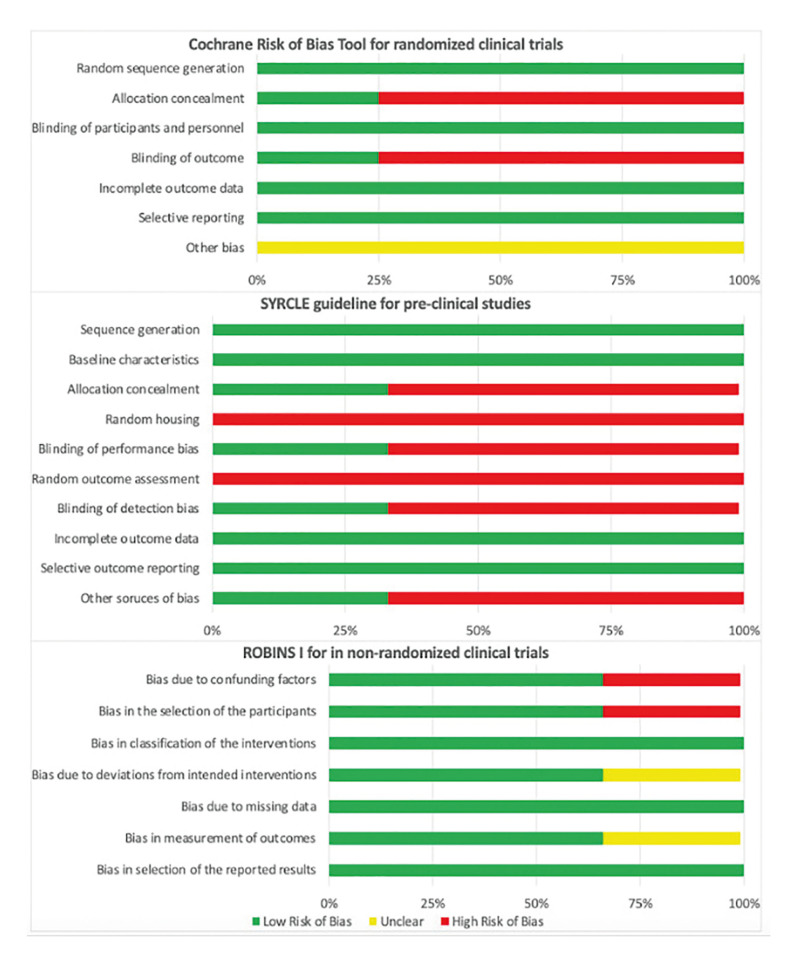
Assessment risk of bias according to study design.

Regarding assessment of the risk of bias (Fig. 3), allocation concealment and blinding the outcome had high risk of bias for randomized clinical trials. For pre-clinical animal studies allocation concealment, random housing, blinding of performance bias, blinding of detection bias and random outcome assessment were not explicitly reported in primary sources.

For non-randomized clinical trials, bias due to confounding factors and bias in the selection of the participants were not described at one article.

Several regenerative approaches were employed, such as resorbable collagen membranes (8,9,20,22), platelet-rich plasma (PRP) (9), platelet-rich fibrin (PRF) (17,18), enamel matrix derivatives (EMD) (21), bone grafts (19,22) and calcium sulfate (10,23).

**Table 1:**
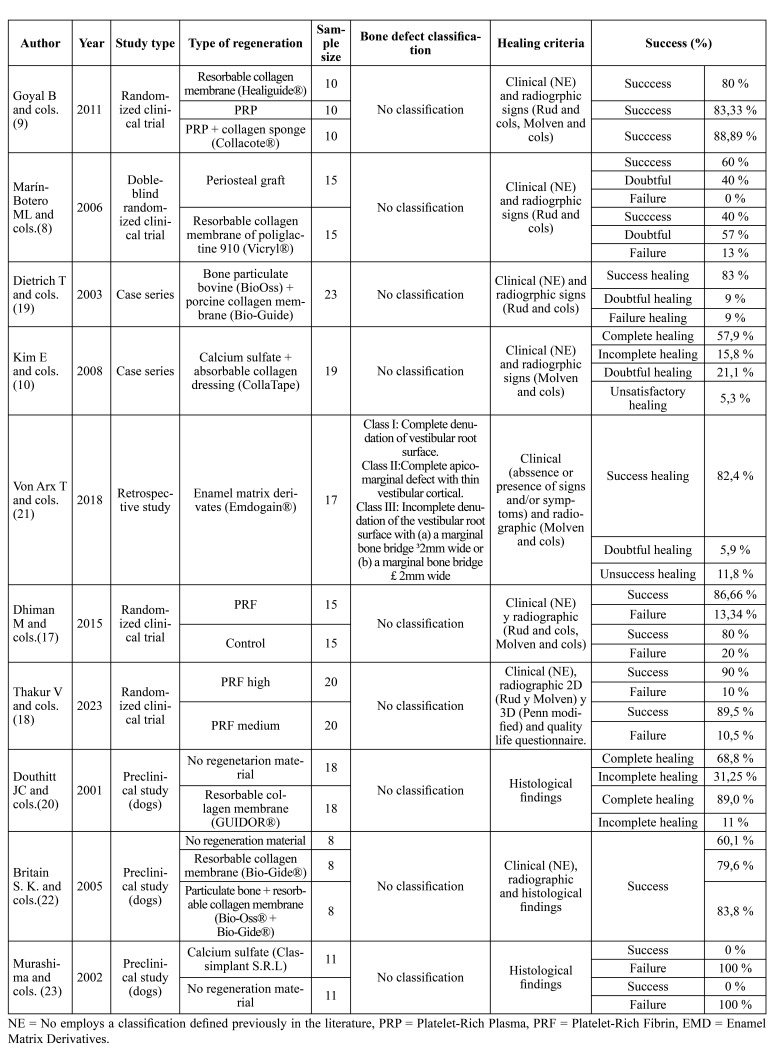
Study type, level of scientific evidence (according to sign's level of evidence for intervention studies), number of cases studied, type of regeneration technique used, pharmacological therapy, defect classification used, healing criterion employed, and percentage of success achieved.

## Discussion

According to this systematic review, the use of regenerative materials in periapical surgery could improve the prognosis of apicomarginal lesions such as resorbable collagen membranes, platelet-rich plasma, platelet-rich fibrin, enamel matrix derivatives, bone grafts and calcium sulfate.

In the context of tissue regeneration for apicomarginal lesions, Platelet-Rich Fibrin (PRF) membranes have demonstrated potential to enhance postoperative outcomes, improving patient functionality and reducing pain perception (24). Presently, PRF can be obtained using various techniques, each resulting in materials with distinct biological characteristics (17). In a recent clinical trial (18), the success rates of using PRF-high and PRF-medium (high or medium centrifugal forces, respectively) were compared, both achieving success rates of approximately 90%. However, when prioritizing patient quality of life, PRF-medium is recommended over PRF-high due to reported reduced swelling and pain in patients treated with PRF-medium.

Goyal *et al*. (9) conducted a study where they achieved clinical and radiographic success rates of 80% in apicomarginal lesion treatment using a collagen membrane, 83.33% by filling the defect with Platelet-Rich Plasma (PRP), and 88.89% with PRP combined with a collagen sponge. All three treatments resulted in statistically significant reductions in probing depth, clinical attachment level, gingival margin position, and periapical lesion size, with no significant differences observed between the three groups for these parameters. However, it's important to note that this clinical trial lacked a control group, which is a common limitation in many human clinical trials on this topic.

In contrast, the PRF study conducted by Dhiman *et al*. (17) included a control group and yielded very similar results. The authors attribute this to PRF acting as a membrane to prevent epithelial migration, serving as a matrix with biological properties such as promoting neoangiogenesis and preventing necrosis and flap shrinkage, in addition to acting as a fibrin glue with space-maintaining capabilities. However, it's essential to exercise caution when extrapolating these findings, as the majority of the lesions in the study had minimal or no periodontal involvement, and the success rate in the control group was very similar.

Experimental research also evaluated regenerative techniques in apicomarginal lesions, primarily in studies with dogs. These studies provide valuable information, they cannot always be directly extrapolated to humans.

A study by Britain *et al*. (22) showed that the use of a collagen membrane (BioGuide®) achieves a success rate of 79.6%, which increases to 83.8% when combined with a bovine bone graft (BioGuide® and BioOss®). These approaches resulted in increased bone, periodontal ligament, and new cementum compared to non-regenerated defects. Douthitt *et al*. (20) used an absorbable collagen membrane (GUIDOR®) in defect regeneration, demonstrating greater alveolar bone regeneration in terms of height and width in the membrane group compared to the control group. This suggested a benefit in using absorbable membranes to reduce patient morbidity. In a more recent study (23), calcium sulfate was used as a regeneration material in simulated apicomarginal lesions in dogs, but no regeneration was achieved in any of the lesions. However, the study noted that factors such as material dissolution in saliva or pus may contribute to the failure.

The present study had some limitations. The classification of apicomarginal defects varied among the studies, and in some cases, a predefined classification was not specified. Various criteria for healing assessment were used, including clinical and radiographic criteria, often modified from previous studies in the field.

## Conclusions

The use of regenerative materials in periapical surgery could improve the prognosis of apicomarginal lesions. Future research in this field should aim to standardize classification and healing criteria to enhance comparability across studies and provide more conclusive evidence for optimal treatment approaches.
